# Chitosan/Poly Vinyl Alcohol/Graphene Oxide Based pH-Responsive Composite Hydrogel Films: Drug Release, Anti-Microbial and Cell Viability Studies

**DOI:** 10.3390/polym13183124

**Published:** 2021-09-16

**Authors:** Muhammad Umar Aslam Khan, Zahida Yaqoob, Mohamed Nainar Mohamed Ansari, Saiful Izwan Abd Razak, Mohsin Ali Raza, Amna Sajjad, Sajjad Haider, Fauzi Mh Busra

**Affiliations:** 1 BioInspired Device and Tissue Engineering Research Group, School of Biomedical Engineering and Health Sciences, Faculty of Engineering, Universiti Teknologi Malaysia, Skudai 81300, Malaysia; saifulizwan@utm.my; 2 Institute for Personalized Medicine, School of Biomedical Engineering, Med-X Research Institute, Shanghai Jiao Tong University, Shanghai 200030, China; 3 Nanoscience and Technology Department (NS & TD), National Center for Physics, Islamabad 44000, Pakistan; 4 Institute of Metallurgy and Materials Engineering, Faculty of Chemical and Materials Engineering, University of the Punjab, Lahore 54590, Pakistan; Zahidayaqoob20@gmail.com (Z.Y.); mohsin.imme@pu.edu.pk (M.A.R.); 5 Institute of Power Engineering, Universiti Tenaga Nasional, Kajang 43000, Malaysia; 6 Centre for Advanced Composite Materials, Universiti Teknologi Malaysia, Skudai 81300, Malaysia; 7 Department of Zoology, Government College University Faisalabad, Faisalabad 38000, Pakistan; amnasajjad7@yahoo.com; 8 Department of Chemical Engineering, College of Engineering, King Saud University, P.O. Box 800, Riyadh 11421, Saudi Arabia; shaider@ksu.edu.sa; 9 Tissue Engineering Centre, UKM Medical Centre, Jalan Yaacob Latiff, Bandar Tun Razak, Cheras, Kuala Lumpur 56000, Malaysia; fauzibusra@ukm.edu.my

**Keywords:** antibacterial activity, biocompatible, chitosan, composite hydrogel, drug release, drug delivery

## Abstract

The composite hydrogels were produced using the solution casting method due to the non-toxic and biocompatible nature of chitosan (CS)/polyvinyl alcohol (PVA). The best composition was chosen and crosslinked with tetraethyl orthosilicate (TEOS), after which different amounts of graphene oxide (GO) were added to develop composite hydrogels. Fourier transform infrared spectroscopy (FTIR), scanning electron microscopy (SEM), atomic force microscopy (AFM) and contact angle was used to analyze the hydrogels. The samples were also evaluated for swelling abilities in various mediums. The drug release profile was studied in phosphate-buffered saline (PBS) at a pH of 7.4. To predict the mechanism of drug release, the data were fitted into kinetic models. Finally, antibacterial activity and cell viability data were obtained. FTIR studies revealed the successful synthesis of CS/PVA hydrogels and GO/CS/PVA in hydrogel composite. SEM showed no phase separation of the polymers, whereas AFM showed a decrease in surface roughness with an increase in GO content. 100 µL of crosslinker was the critical concentration at which the sample displayed excellent swelling and preserved its structure. Both the crosslinked and composite hydrogel showed good swelling. The most acceptable mechanism of drug release is diffusion-controlled, and it obeys Fick’s law of diffusion for drug released. The best fitting of the zero-order, Hixson-Crowell and Higuchi models supported our assumption. The GO/CS/PVA hydrogel composite showed better antibacterial and cell viability behaviors. They can be better biomaterials in biomedical applications.

## 1. Introduction

With increased environmental awareness and an emphasis on eco-friendly products, the goal of research efforts is in the production of biocompatible, biodegradable and low-cost film-forming materials for biomedical applications [1,2,3]. Due to their biocompatibility, nontoxicity, biodegradability, ease of availability and low cost, natural polymers have played an important role in producing biomaterials. CS, a natural biopolymer, is regarded as an outstanding candidate for film formation [4]. Alkaline de-acetylation is used to obtain a naturally occurring biopolymer (CS) with a poly-cationic structure from the chitin shells of shrimp and other crustaceans. It’s a polysaccharide made up of *N*-acetyl d-glucosamine and d-glucosamine units linked by a (1–4) glycosidic bond that’s abundant in nature [5]. CS has been widely used in biomedical and drug delivery applications. It possesses biocompatibility, low toxicity, mucoadhesive properties, antibacterial activities and permeation-enhancing characteristics [5]. However, on the other hand, CS has low mechanical properties that limiting its applications. Several methods have recently been developed to improve the mechanical properties of CS by using nanofillers, for instance, graphene and their derivatives [6]. GO is commonly used in biomedical applications and other carbon-based nanomaterials such as carbon nanotubes (CNTs). It has unique chemical properties, including such as high biocompatibility, low toxicity, a large surface area for effective drug binding, oxygen-containing functionalities and improved conductivity [7]. GO is graphite-derived two-dimensional (2D) carbon material. It has a long history of use as a precursor to chemically converted graphene. Many hydrophilic oxygenated functional groups in GO, such as hydroxyl (–OH), epoxy (–C–O–C–) and carboxyl (–COOH), enable it to disperse in water solution and be easily exfoliated into monolayer sheets [8]. Due to these functional classes, GO is amphiphilic with hydrophilic edges and hydrophobic basal planes. GO contains oxygen-enriched functional groups, which react with various polymers to form polymer-based graphene oxide nanocomposite [9,10]. The large surface area of GO is used to carry and distribute drugs through contact between GO functionalities and drug groups. The importance of GO-based hydrogels in drug release is well known [11], and GO-based drug delivery systems have been extensively researched due to their pH-sensitive drug release behavior. By adjusting the pH of the GO-based hydrogel, controlled drug release was achieved [12]. Polyvinyl alcohol (PVA) is known as green material due to its nontoxicity and biocompatibility. PVA is biodegradable, chemically stable, biocompatible, eco-friendly, as well as water-soluble. It has been extensively researched in various fields and has several available hydroxyl groups (OH) on its polymeric chain [13]. As a result, PVA-based hydrogels find use in the pharmaceutical industry for managed drug delivery. Kim and Kim et al. [14] studied the relationship between the percentage of PVA and drug release time. They developed double-layered composite PVA beads for managed drug delivery. They found that as the amount of PVA increased, the release of therapeutic agents was delayed due to increased cross-linking. Crosslinking is used to enhance the resistance of hydrogel to disintegration in any solution [15]. TEOS is well-known cross-linker and has been widely used in biomedical applications. Several cross-linkers (TEOS, formaldehyde, acetaldehyde and glutaraldehyde, etc.) have been used to cross-link different polymeric chains to develop biomaterials. However, TEOS is an ideal cross-linker since it provides covalent bonding between inorganic and polymer chains [16].

Combining different polymers as a blend is a valuable strategy to create new materials with low cost and synergy in properties. For instance, CS/GO-based hydrogel has been developed without the addition of cross-linker by non-covalent interactions for the application of rapidly self-healing performance. The GO contains several oxygen-based functional groups that formed hydrogen bonds with an amino group of CS to improve the mechanical properties of hydrogel [17]. PVA/CS hydrogel is reported in the literature for its biomedical applications since it has appropriate biocompatibility and nontoxicity. Flexible and robust PVA/GO composite films were prepared with a layered structure by vacuum filtration [18].

Similarly, CS/PVA blended films cross-linked via glutaraldehyde for skin tissue repair are also reported in the literature. These hydrogels have potential applications in artificial muscle and controlled drug release [19]. Various research studies have demonstrated the excellent pH-sensitive ability of PVA and GO. These CS/GO composites films have other widespread applications for bone tissue engineering, drug delivery and water treatment [20]. CS/GO becomes a stable and biocompatible composite with excellent thermal and mechanical properties. Due to the strong hydrogen bonds and electrostatic attraction between negatively charged GO sheets and positively charged polysaccharides groups in CS. Under proper pH conditions, uniformly dispersed CS/ GO films could be fabricated. It is known that the amino group of CS interacts with oxygen functionalities of GO through hydrogen bonding, which results in improved mechanical properties [21].

In this work, a series of hydrogels were synthesized by blending the PVA/CS. The performing samples were selected for the suspension of GO. The prepared hydrogels were characterized by Fourier transform infrared spectroscopy (FTIR), Scanning electron microscope (SEM), Atomic Force Microscope (AFM) and contact angle. The hydrogels were also tested for their swelling capabilities in different media. The drug release profile was investigated in phosphate buffer saline (PBS) at pH = 7.4. Finally, antibacterial activity and cell viability data were obtained.

## 2. Materials and Methods

### 2.1. Materials

Chitosan (CS), Tetraethoxysilane (TEOS) and Polyvinyl Alcohol (PVA) were purchased from Merck, Darmstadt, Germany. Graphite powder, NaCl, Na_2_HPO_4_, KH_2_PO_4_, KCl, NaOH, HCl, Acetic acid, Phosphoric acid, Sulphuric acid, Potassium Permanganate (KMnO_4_), Hydrogen peroxide (H_2_O_2_) and ethanol were purchased from Sigma-Aldrich, Burlington, Massachusetts, USA. Deionized water was used for the preparation of solutions and suspensions. Paracetamol was obtained from a local pharmaceutical company. Mouse pre-osteoblast (*MC3T3-E1*) cell lines were purchased from the American Type Culture Collection (ATCC-USA), and Hyclone Laboratories Inc, Logan, Utah, USA. supplied Alpha-MEM (α-MEM). The fetal bovine serum (*FBS*) and l-glutamine penicillin/streptomycin were obtained from ThermoFisher Scientific, Gloucester, United Kingdom.

### 2.2. Experimental Details

#### 2.2.1. Synthesis of Graphene Oxide

GO was prepared by a well-known Hummer method [22]. Graphite powder was used as a precursor for the synthesis of GO. Briefly, 3 g of graphite powder was added to the mixture of H_3_PO_4_/H_2_SO_4_ (1:9). Then, 18 g of KMnO_4_ was added slowly to the mixture under continuous stirring for 12 h using a hotplate. When the reaction is completed, deionized water (400 mL) was added to the mixture, and the mixture was titrated with 3 mL (30% H_2_O_2_). Finally, the solution was filtered using filter paper, and the filtrated residues were washed with 30% HCl, ethanol, and deionized water repeatedly until neutral pH 7. The resultant residues were dried at 60 °C for 12 h in a vacuum oven and stored in sample containers [23].

#### 2.2.2. Synthesis of Hydrogels

A series of CS/PVA blended films were fabricated via solution casting technique. CS (0.6 g) was dissolved in 2% acetic acid, and PVA (0.4 g) was dissolved in 50 mL deionized water. CS and PVA solutions were mixed under continuous stirring at 55 °C for 2 h. TEOS (100 µL) dissolved in10 mL ethanol was added dropwise into the stirring homogeneous solution at 55 °C. The stirring was continued at 55 °C for another 3 h. After that, the solution was poured carefully into well-cleaned and dried Petri dishes. The Petri dishes were kept in the oven at 50 °C for 3 days. The films with 100 and 150 µL TEOS were prepared via repeating the same procedure. The 100 and 150 µL were coded as CP-1, CP-2. The controlled film sample was synthesized using the same procedure but without the addition of TEOS. The controlled sample was coded as CP. The formulation is tabulated in Table 1.

##### Formulation of CS/PVA/GO Hydrogels

**Figure 1:**
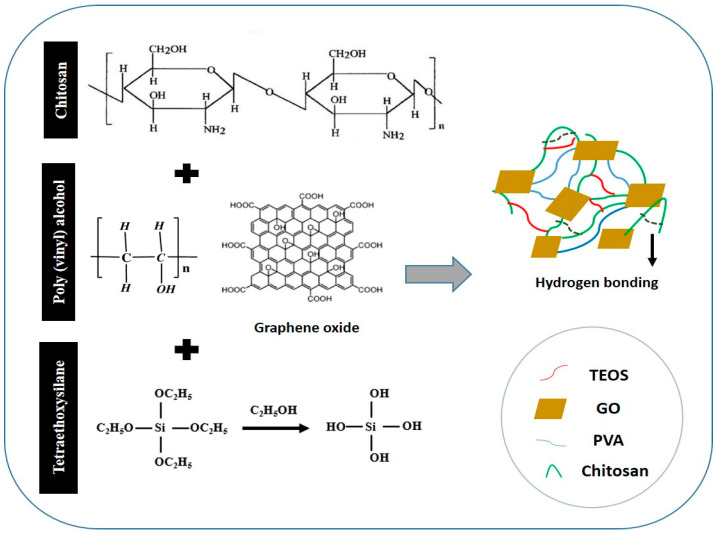
Proposed chemical mechanism of chitosan/polyvinyl alcohol/Graphene oxide composite films.

**Figure 2:**
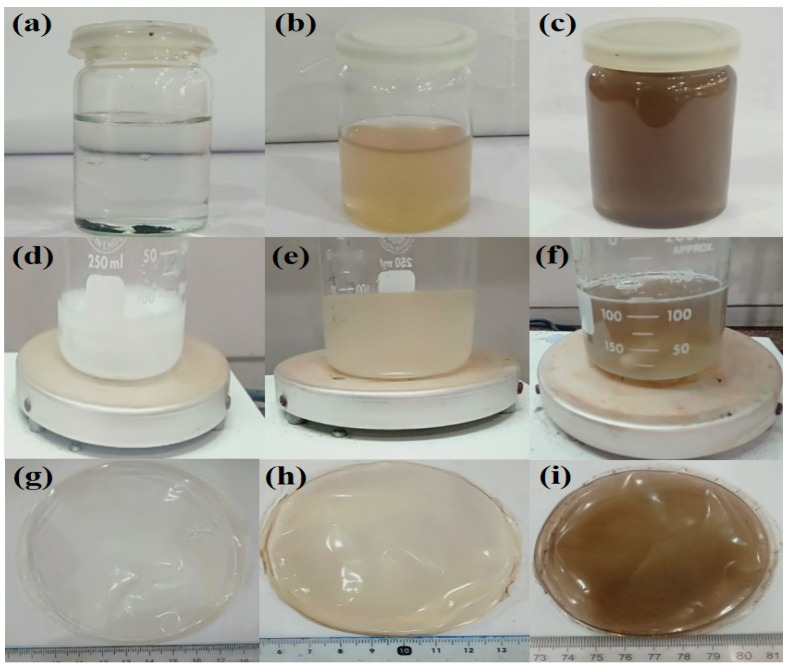
Photographs of CS/PVA/GO based hydrogel (**a**) graphite oxide in glass vial before dispersion, (**b**) 0.1% dispersed GO in aqueous solution, (**c**) 0.5% dispersed GO, (**d**) CP-1 solution, (**e**) CPGO-0.1%, (**f**) CPGO-0.5%, (**g**) CP-1 composite film, (**h**) CPGO-0.1% composite film and (**i**) CPGO-0.5 % composite film.

0.1 % and 0.5% of the prepared GO was dispersed via ultrasonication in deionized water (DI) until it was completely dispersed. The homogeneously dispersed solution of GO had light brown and dark brown colors (Figure 2b,c). Composite films with a varying weight ratio of GO (0, 0.1% and 0.5%) were fabricated via the solution casting technique. The proposed chemical mechanism has been presented in the schematic diagram. The samples were coded as CP-1, CPGO-0.1% and CPGO-0.5%. The proposed mechanism of the fabrication of the hydrogels and GO/ hydrogel composite is depicted in Figure 1, and the digital photographs of composite films are shown in Figure 2g–i. The formulation is tabulated in Table 1.

#### 2.2.3. Drug-Loading into CS/PVA/GO Hydrogels

CS, PVA and GO solutions were prepared and then blended as mentioned in Section 2.2.2. After 1 h of blending, 20 mg of paracetamol dissolved in 10 mL deionized water was added dropwise to the blended solution by a dropper. The solution was further stirred for 1 h until all the drugs become dissolved. 100 µL of TEOS was dissolved in 10 mL of ethanol and was added dropwise into the drug-loaded blend. The final blend was further stirred for 3 h and poured carefully into the well cleaned and dried Petri dish. The casted films were kept in a vacuum drying oven at 55 °C for two or three days.

## 3. Characterization

### 3.1. Fourier Transform Infrared Spectroscopy (FTIR)

The functional group and interactions among all of the components of the prepared hydrogels were investigated using FTIR spectroscopy in ATR mode (Agilent Cary 630). The FTIR study was carried out at a scan rate of 60 scans and wavenumber ranging from 4000 to 400 cm^−1^, with 4 cm^−1^ resolutions.

### 3.2. SEM Morphology

Scanning electron microscopy (SEM, JEOL-JSM-6480, Akishima, Japan) was used to examine the morphologies of the well-dried hydrogels, and hydrogel films were sliced and gold-sputtered. These were placed on a stub before being placed in the vacuum chamber to observe surface morphology.

### 3.3. Swelling and Degradation Analysis

The swelling experiments of the hydrogel (CS/PVA) and the composite (CS/PVA/G0-0.1% and CS/PVA/GO-0.5%) were conducted in DI water, buffer and salt solutions. The blended dried films were first to cut into small pieces, dried and weighed. Then, the samples were placed in DI water in separate containers and allowed to swell. The samples were removed from the containers at pre-decided intervals, adequately cleaned with filter paper to remove the surface water, and weighed again. The swelling experiments were conducted in DI, Buffer solution (at pH 2, 4, 7 and 10) and electrolyte solution (NaCl and CaCl_2_ (0.1 M, 0.3 M, 0.7 M and 0.9 M). Swelling (DS) was calculated using Equation (1) [24]. The swelling analysis was performed in triplicated, and average values were taken to calculate the swelling analysis.

(1) $$\mathrm{Swelling}\;\mathrm{index}=\frac{Ws-Wd}{Wd}$$

where *Ws* = swollen weight of films, and *Wd* = the dried weight of the films.

The well-dried hydrogels films were cut into square form and weighted (45 mg) carefully. The degradation analysis was performed in PBS solution with pH = 7.4, and these samples were incubated at 37 °C for different periods (1, 2, 3, 5 and 7 days). The degradation of hydrogels was calculated using Equation (2). [24]. The degradation analysis was performed in triplicates, and average values were taken to calculate degradation analysis.

(2) $$\mathrm{Weight}\;\mathrm{loss}\;(\%)=\frac{W_{0}-W_{t}}{W_{0}}\times 100$$

where *W*_0_ = initial hydrogel weight, and *W_t_* = hydrogel weight at the time “*t*”.

### 3.4. Atomic Force Microscopy (AFM)

AFM (Nano-Solver, NT-MDT) equipped with a silicon nitride tip was used to investigate the surface roughness of the hydrogel samples at ambient conditions. AFM was performed using Nova-Px software over an area of 5 μm × 5 μm in the semi-contact mode. Dried samples (thin films) were used and stuck to the sample holder for AFM analysis.

### 3.5. In-Vitro Drug Release Procedure

The dried drug-loaded hydrogel was immersed into the beaker containing 100 mL of simulated PBS (pH = 7.4), composed of ions with concentrations close to those of ions present in human blood plasma. The beaker was placed on a magnetic stirrer to maintain the temperature of the system at 37 °C. The samples were withdrawn at various time intervals such as 10, 20, 30, 40, 50 and 60 minutes by syringe, and an equal volume of fresh dissolution medium of PBS pH 7.4 was replaced after each sampling. The sampling process was continued until 180 minutes to evaluate drug release behavior. At the above-mentioned periodic intervals, the drug content in the sample was analyzed at 243 nm in the UV- visible spectrophotometer (Spectro-115U, Reference 3000, Gamry Instruments, Warminster, PA, USA) against reference standard (PBS pH 7.4 as a blank). The amount of the drug was calculated using a standard working curve.

### 3.6. Contact Angle Measurement

The contact angle measurement was performed via a contact angle analysis system (JY-82, Dingsheng, Chengde, China). Water contact angle (θ) was measured for each sample (1 μL with a flow rate of 0.1 μL/s), and contact angle values were recorded within 0–5 s after placing the drop. The average contact angle values were obtained at least 10 drops in various areas to test the surface, and three test surfaces for each condition were used. All measurements were conducted in air and at ambient temperature.

### 3.7. Antibacterial Activities

The antimicrobial activities of composite hydrogels were observed against the Gram +ive *Staphylococcus aureus* (S. A.) and Gram -ive Escherichia coli (E. C.) by a well-reported method [22]. A disc diffusion method was employed by placing 80 μL on agar plates. Zone inhibition was measured in mm after incubation for 24 h. After 24 h, the circular inhibition zones were formed and photographed with no bacterial growth.

### 3.8. In-Vitro Studies

#### 3.8.1. Cell Culture and Morphology

The pre-osteoblast (*MC3T3-E1*) cell lines were maintained into *α-MEM* without ascorbic acid containing 10% FBS, 1% (2 mM) L-glutamine, 1% penicillin/streptomycin. The cell density was assumed to be 5000 cells/cm^2^ in a 100-mm culture plate. At the same time, gelatin (0.1% by conc.) was employed as a coating agent and controlled. These cell lines with hydrogels were incubated under standard in-vitro conditions (37 °C, 5% CO_2_ and 90% humidity). 

#### 3.8.2. Cell Viability

*MC3T3-E1* cell lines were cultured in varying concentrations of hydrogels (0.500, 1.000 and 2.000 mg/mL), 0.1% gelatin (+ive control) and 1% DMSO (–ive control) and incubated under standard in-vitro conditions for 72 h. After 72 h, these cultured cells were treated with a neutral red agent by a well-reported method by Repetto et al. [25]. These experiments were performed in triplicate, and these treated cells were incubated in a neutral red medium (40 μg/mL) for 2 h. The excess neutral red stain was removed by washing these cells with PBS solution and incubating for 2 h. Later, these cell lines were treated with destaining solution (50% distilled water, 49% absolute ethanol and 1% glacial acetic acid) at 37 °C for 10 min to destained cell lines. An absorbance microplate reader observed the optical density was observed at 570 nm by an absorbance microplate reader (Bio-Tek, ELx-800, Winooski, VT, USA). The cell viability percentage was calculated by Equation (3).

(3) $$\mathrm{Cell}\;\mathrm{viability}=\frac{OD_{S}}{OD_{C}}\times 100$$

whereas; *OD_S_* = optical density of sample concentration and *OD_C_* = the optical density of the positive control.

### 3.9. Statistical Analysis

The obtained experimental data has been presented in mean and standard errors (mean ± *S.E.*), and it is calculated by statistical software (IBM, SPSS Statistics 21). The mean, standard errors of means have been shown as Y-error bars in figures. (*p* < 0.05; n = 3).

## 4. Results and Discussion

### 4.1. Morphology Study

**Figure 3:**
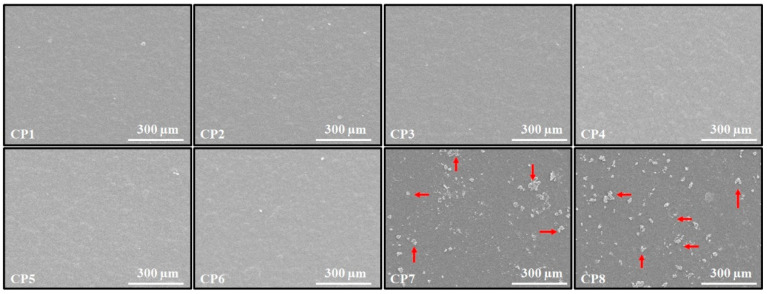
Surface SEM micrographs of the CS/PVA and CS/PVA/GO hydrogels film.

The surface SEM images of the CS/PVA and CS/PVA/GO hydrogels are shown in Figure 3. The SEM micrographs indicate that all of the CS/PVA hydrogels have homogenous surfaces; this indicates that both polymers mixed effectively. Furthermore, not a single hydrogel displayed phase separation when the CS to PVA ratio was changed, confirming that concentration changes did not affect the miscibility of the two polymers. Surfaces of CS/PVA/GO hydrogels indicated the presence of an exterior phase. This exterior phase comprises GO particles that have grown from the hydrogel surface [26].

### 4.2. Swelling and Degradation Analysis

**Figure 4:**
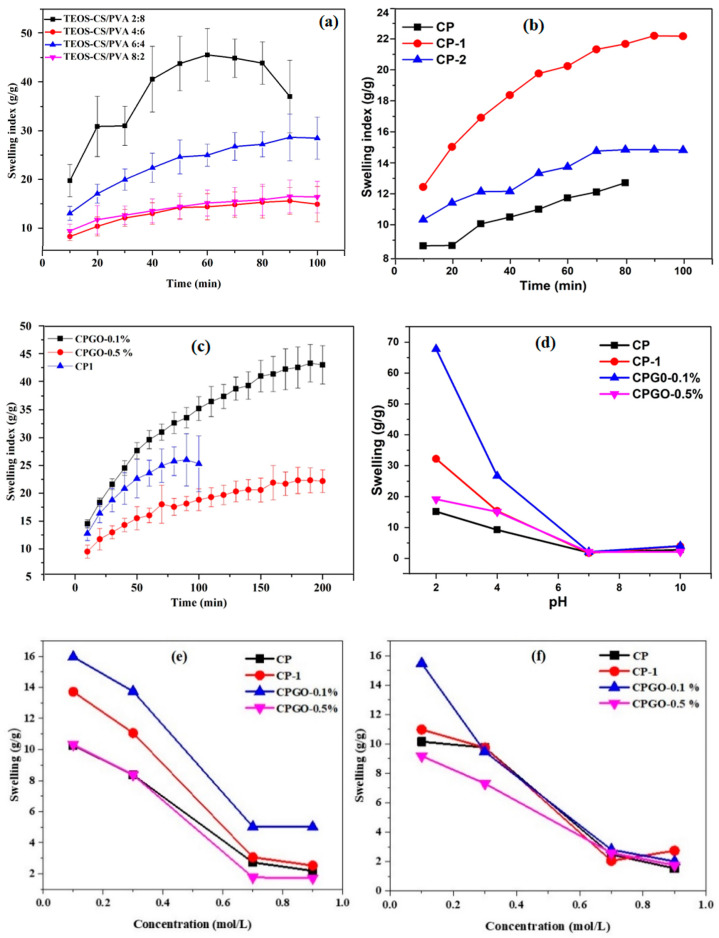

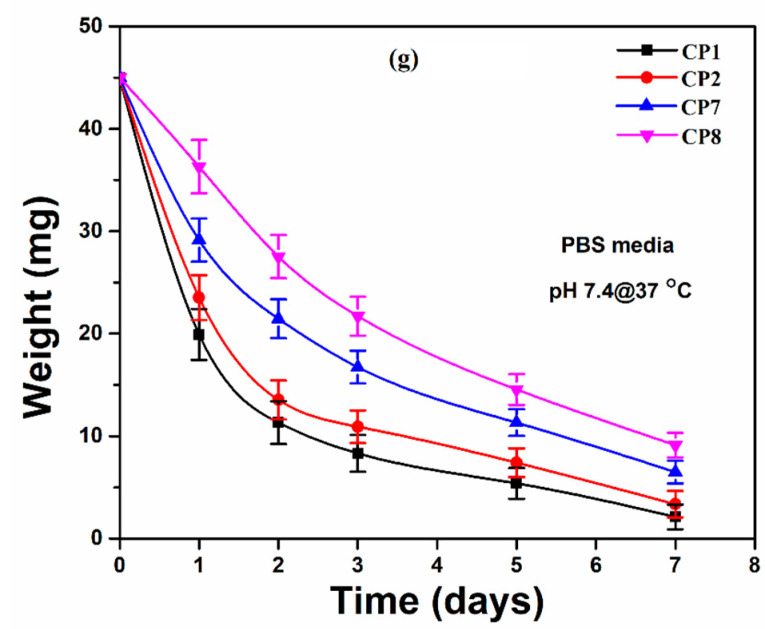
Swelling behavior of the hydrogels as a function of polymer ratio, cross-linker concentration, different GO amount, different pH, salt concentration and degradation (**a**) polymers ratio, (**b**) cross-linker concentration, (**c**) different GO amount, (**d**) different pH, (**e**,**f**) salt concentration and (**g**) degradation analysis of hydrogel films in PBS media (pH = 7.4 at 37 °C).

Figure 4a–f illustrates the hydrogels’ swelling behavior as a function of polymer ratio, cross-linker concentration, GO concentration and salt concentration and type. As shown in Figure 4a, as the ratio of the polymers in the hydrogel was changed, keeping the cross-linker constant, swelling is increased. The maximum swelling was obtained at CS/PVA (2:8). It was also found that the sample was unstable in the aqueous solution and was challenging to handle. It can be easily observed by the large error bars. The sample’s instability is attributed to the fact that just one polymer, CS, is cross-linked, resulting in a loosely bounded semi-interpenetrating network (semi IPN). As the CS contraction in the hydrogel increased, the hydrogel became more stable. The swelling and stability of the hydrogels were optimized by keeping ratio 6 (CS): 4 (PVA), and the relationship between stability and swelling behavior of the hydrogel was investigated. The swelling behavior of a hydrogel with the same polymer ratio, with different cross-linker concentrations (100 and 150 L), was investigated further. Figure 4b clearly shows that the sample without cross-linking did not exhibit proper swelling behavior, but the hydrogel sample with a 100 µL cross-linker demonstrated optimal swelling. It was demonstrated that 100 µL of cross-linker was the critical concentration since the sample displayed excellent swelling and preserved its structure. The decrease in the swelling of hydrogels (with a cross-linking concentration of 150 μL) can be ascribed to the contraction of pore size and tremendous interconnectivity, which may explain why water was unable to enter the spaces, resulting in the decrease in swelling. This tendency is further verified and supported by reducing swelling as the cross-linker quantity has increased. Similar results were also obtained by Wang et al. [22]. The cross-linked hydrogel having CS/PVA 6:4 was selected for the fabrication of the composite hydrogels.

The effect of GO content on hydrogel swelling is depicted in Figure 4c. The swelling ratio of hydrogels with the maximum GO concentration tends to be lower than CP-1 and CPGO-0.1%. This result is attributed to the increasing cross-linking density of hydrogel due to the hydrogen bonds between GO, CS and PVA, which act as a physical cross-linker [27]. As shown in Figure 4d, the hydrogels were also exposed to varied pH (2, 4, 7 and 10) conditions, which resulted in different swelling behaviors. The pH of the swelling media affects the swelling ratio, which can be ascribed to the amino groups in the hydrogel network structure. In an acidic environment, CPG0–0.1% swells more than CP, CP-1 and CPG0–0.5%. The significant swelling in acidic conditions is due to the charged amino groups (NH^3+^) in the polymeric structure, which causes electrostatic repulsion between the polymer chains, enabling maximal water absorption in the network structure [28]. Deprotonation of the amine functional groups occurs in a neutral or alkaline media, and the NH^3+^ groups return to NH_2_. These hydrogels are pH-sensitive due to a carboxylic group of graphene oxide and amino groups of CS. Figure 4e,f shows two trends for CP, CP-1, CPGO-0.1% and CPGO-0.5% in varied molar concentrations of NaCl and CaCl2 solutions. The swelling ratio of hydrogels reduced as the concentrations of both electrolytes increased. All hydrogels displayed better swelling at 0.1 mol/L NaCl and CaCl_2_ concentrations. The formation of ion pairs is responsible for reducing swelling of the hydrogels as the salt concentration increases. Ion pairs act as new cross-linking sites, which causes the polymer chain to shrink and decreasing swelling (24 h).

The degradation of hydrogel films was conducted in PBS media to determine the degradation behavior of well-dried hydrogel films. It was observed that hydrogel samples CP1 and CP2 degraded faster than the hydrogel samples CP7 and CP8. All these hydrogel samples have the same amount of TEOS (crosslinkers). However, hydrogel samples CP1 and CP2 are without GO. Even the degradation is different for the hydrogels sample CP7 and CP8. It is attributed to the different amounts of GO in these samples. Hydrogel sample CP7 with less amount of GO degraded more than CP8. Hence, different degradation behavior of the hydrogel sample confirms that they are successfully crosslinked and have developed differently types of interaction due to GO in hydrogels sample CP7 and CP8. Therefore we can say that an increasing the amount of GO may cause a delay in the degradation of the hydrogel samples.

### 4.3. FTIR Analysis

**Figure 5:**
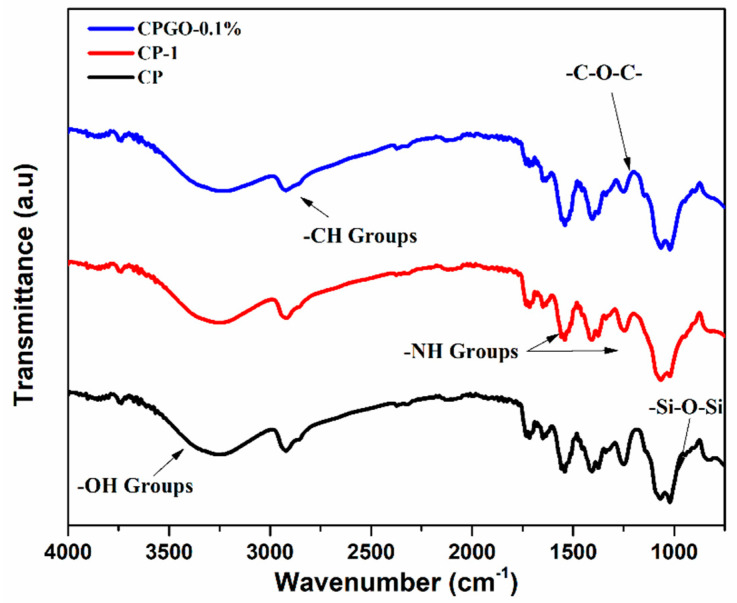
FTIR spectra of CS/PVA and CS/PVA/GO hydrogels.

FTIR spectrum of different samples was investigated, as shown in Figure 5. The functional groups confirmed the oxidation process on the GO surface. The FTIR spectrum of CPG0–0.1% displays a broadband between 3212 to 3258 cm^−1^, which showed –OH stretching of intermolecular and intramolecular hydrogen bonding. The characteristic peaks of alkyl groups (–CH stretching) were observed at 2920 cm^−1^. The peak at 1246 cm^−1^ was attributed to –C–O–C stretching [29]. The peaks between 1400 cm^−1^ to 1500 cm^−1^ which attributed to –NH stretching. The FTIR spectrum of CS/PVA hydrogels revealed the characteristic peaks of siloxane at 1065 cm^−1^. The FTIR investigations confirmed the presence of siloxane linkage among chitosan, polyvinyl alcohol and GO through the intermolecular hydrogen bond formation along with TEOS [30].

### 4.4. AFM Topography

**Figure 6:**
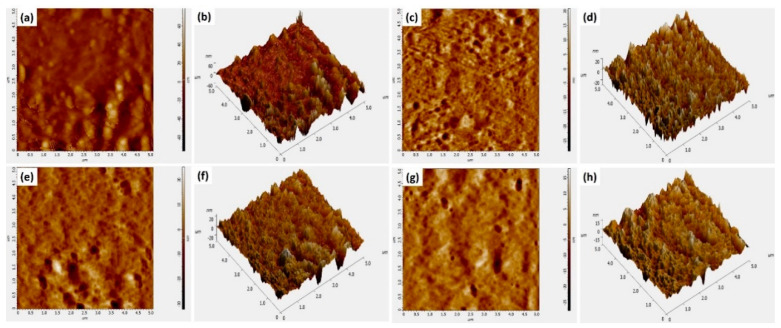
AFM images of (**a**,**b**) CP-1, (**c**,**d**) CP-2 (**e**,**f**) CP-7 and (**g**,**h**) CP-8.

AFM images of the prepared hydrogels (Figure 6) show a fascinating behavior in terms of surface roughness. The controlled film without cross-linker (CP) shows the highest roughness as compared to other samples. It is well understood as the non-crosslinked sample has a relatively open and porous structure. Adding GO in the range of 0.1 to 0.5 µg/mL to the cross-linked hydrogel (CP-1) causes the roughness of hydrogel samples to decrease gradually (Table 2). This reduction in roughness is due to the large surface area of GO which covers the pores, and a more compact structure of the hydrogel that offers a smooth surface morphology [31].

### 4.5. In Vitro Drug Release Analysis

**Figure 7:**
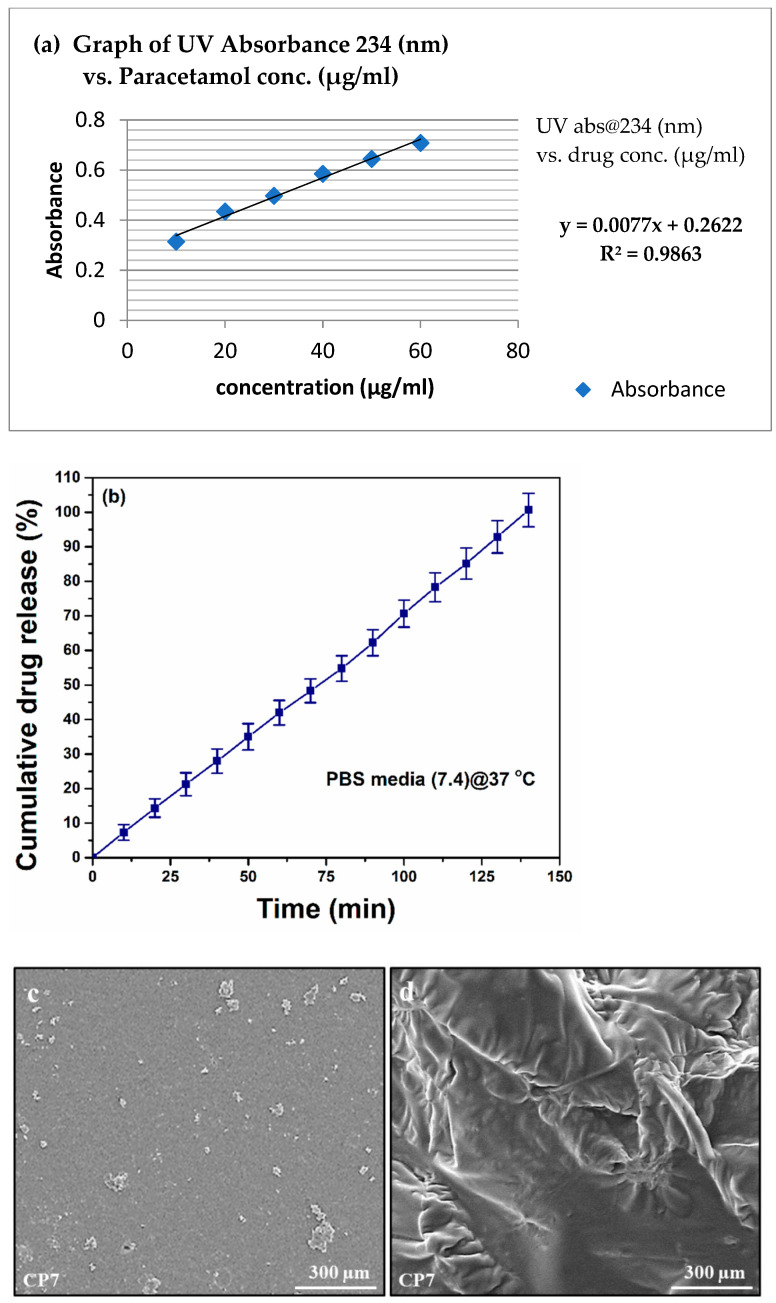
(**a**) Calibration curve of paracetamol in PBS pH 7.4 (**b**) Cumulative percentage of drug release with time (min) for CP7 hydrogel, (**c**) SEM analysis of C7 before drug release and (**d**) SEM analysis of CP7 after drug release.

Hydrogels offer different release mechanisms to entrap drugs, such as swelling controlled, chemically controlled and diffusion-controlled mechanisms. The most acceptable one is the diffusion-controlled mechanism, and it obeys Fick’s law of diffusion for drug released [32]. The diffusion coefficient of the hydrogels is related to the porosity of the hydrogels if the pore size of porous hydrogels is greater than the molecular dimensions of the drug molecule. Drug release only follows the swelling controlled mechanism if drug release time exceeds the time of swelling [21]. The release behavior of paracetamol from the hydrogel with and without GO is shown in Figure 7. The paracetamol releases slowly from GO, and the release rate gradually declines after 140 min. Figure 7c,d presents the SEM analysis of CP7 to determine surface morphology before and after drug release analysis. 

On the other hand, CP1 released 90% drug within 120 min. The sustained release of drug in CPGO-0.1% attributed to the incorporation of GO in the polymer matrix. A six-point calibration graph was constructed in the concentration range of 10–60 µg/mL to accurately measure the released drug amount. Paracetamol drug release kinetic studies were investigated against various models (zero-order, first-order, Higuchi, Korsmeyer-Peppas and Hixson-Crowell, models) [21,32]. The mathematical model proves to be highly effective in forecasting drug release and predicting the mechanism of release.

(4) $$\text{Zero-order}\;M_{t}=M_{o}+K_{o}t$$

(5) $$\mathrm{First}\;\mathrm{order}:\;logC_{o}-\;\frac{kt}{2.303}$$

(6) $$\mathrm{Higuchi}\;\mathrm{model}:\;ft\;=Q=K_{H}\;\times \;t^{1/2}$$

(7) $$\mathrm{Hixson}\;\mathrm{Crowell}\;\mathrm{model}:\;W^{1/3}-W^{1/3}=\;kt$$

(8) $$\text{Korsmeyer-Peppas}\;\mathrm{model}:\;ln\frac{M_{t}}{M_{o}}\;=n\;lnt\;+lnK$$

whereas *M_t_* = drug release amount at time *t*, *K_H_*, *K* and *Ko* are constants.

**Figure 8:**
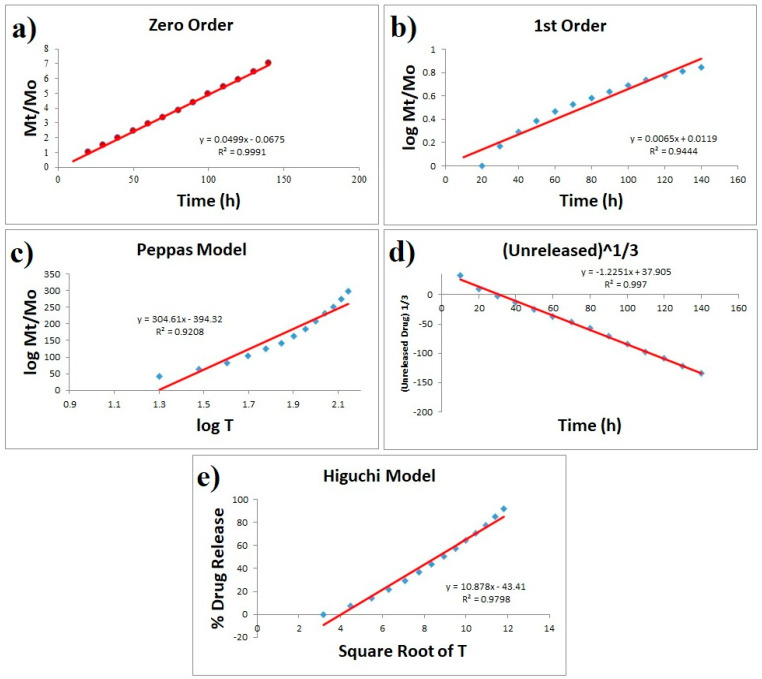
Paracetamol release drug studied various kinetic models ((**a**) zero-order, (**b**) first-order, (**c**) Higuchi, (**d**) Korsmeyer-Peppas and (**e**) Hixson-Crowell).

To explain the dissolution profile, model-dependent approaches use several mathematical functions. To investigate the drug release mechanism of paracetamol, model-dependent methods such as zero order, first order, Higuchi, Hixson-Crowell and Korsmeyer-Peppas models were used. Figure 8 clearly shows that best fitting was obtained with zero order, followed by Hixson-Crowell and Higuchi models. The zero-order model says that the dissolution of dosage forms does not disaggregate and slowly releases a constant drug. According to Hixson-Crowell, drug release is a process that is governed by diffusion, dissolution or both. Generally, diffusion-controlled drug release is predicted by the Higuchi square root of time model. Higuchi model is based on the hypotheses that (i) initial drug concentration in the matrix is much higher than drug solubility; (ii) drug diffusion takes place only in one dimension (edge effect must be negligible); (iii) matrix swelling and dissolution are negligible; (iv) drug diffusivity is constant; and (v) in the release environment, ideal sink conditions are always achieved. Since our results are obeying all three models (Figure 8) and since all of these models are very close when it comes to diffusion; hence it can be concluded that the release of paracetamol from the present system is a controlled process that is unaffected by drug concentration [21,33,34]. 

### 4.6. Water Contact Angle Measurement

**Figure 9:**
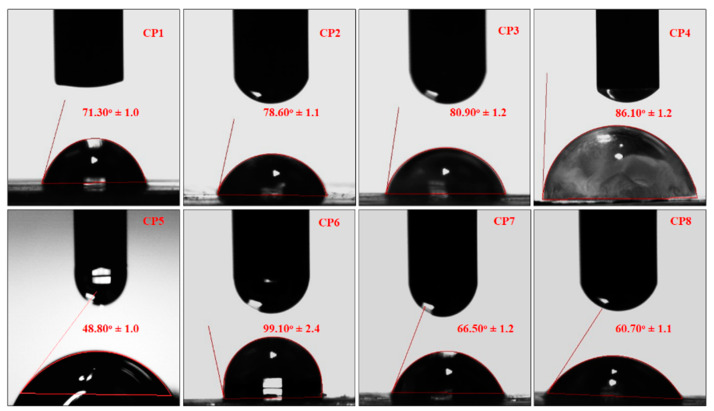
The wetting behavior of all samples of hydrogels.

The water contact angle is vital for determining hydrogel hydrophilicity or hydrophobicity, an essential factor in drug delivery, cell proliferation and adherence. Consequently, the wetting behavior of hydrogel samples (CP1, CP2, CP3, CP4, CP5, CP6, CP7 and CP8) was analyzed using water contact angles of hydrogels. The water contact angle is vital for determining hydrogel hydrophilicity or hydrophobicity, an essential factor in drug delivery, cell proliferation and adherence [33]. TEOS cross-linking played a crucial role in studying the water contact angle, as seen in Figure 9. The increasing amount of PVA shifts wetting behavior from hydrophilicity to hydrophobicity with an optimized quantity of cross-linker [34].

In comparison, contact angle values, different behavior of CP7 and CP8 were observed due to different amounts of GO sheets. F8 is more hydrophilic than CP7 due to the amount of GO sheet [35]. The hydrophilic behavior of hydrogels (CP7 and CP8) issue to various functional groups available over the surface that encourages Hydrogen bonding. The hydrogel samples showed improved water contact angle values due to the increasing amount of TEOS quantities. The increasing cross-linking changes the wetting behavior of hydrophilicity to hydrophobicity [36,37]. Since the close packing of polymeric structure and free functional are not available due to more covalent bonding, very few functional groups are available for hydrogen bonding and increasing cross-linking due to the increasing amount of TEOS, the hydrophilic character was shifted towards hydrophobic character [38]. Hence, hydrophilicity can be optimized using appropriate TEOS for required cross-linking for controlled drug delivery, swelling of hydrogel cell proliferation and adherence. 

### 4.7. Antimicrobial activity

**Figure 10:**
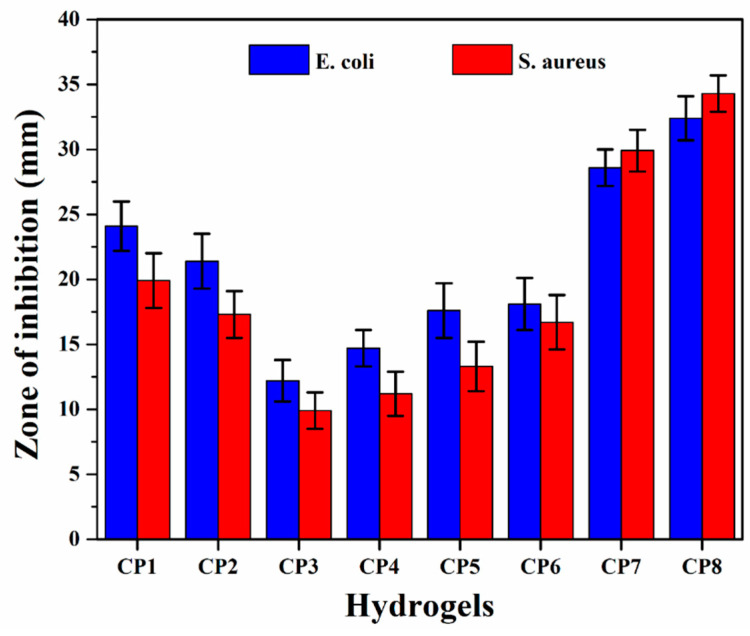
Antibacterial activities of all samples of hydrogels against severe disease-causing pathogens.

The antimicrobial activities of all hydrogels are shown in Figure 10—the antimicrobial activities against Gram +ive and –ive bacterial *S. aureus* and *E. coli* were studied using the agar well diffusion method. The hydrogel samples with different compositions demonstrated different inhibition zones (mm) against the used pathogens. The hydrogel samples with GO (CP7 and CP8) showed maximum antibacterial activity, whereas those without GO (CP1, CP2, CP3, CP4, CP5 and CP6) showed lower antibacterial activity. Both the increase in CS and GO in the hydrogels enhanced the antibacterial activities. The different zones of inhabitations against *E. coli* for samples were CP1 = 19.9 ± 1.0, CP2 = 17.3 ± 1.2, CP3 = 9.9 ± 1.1, CP4 = 11.2 ± 1.1, CP5= 13.3 ± 1.2, CP6 = 16.7 ± 1.3, CP7 = 29.9 ± 1.3 and CP 8 = 34.7 ± 1.5 and against *S. aureus* were CP1 = 24.1 ± 1.4, CP2 = 21.4 ± 1.2, CP3 = 12.2 ± 0.90, CP4 = 14.7 ± 1.0, CP5 = 17.6 ± 1.2, CP6 = 18.1 ± 1.1, CP7 = 28.6 ± 1.2 and CP8 = 32.4 ± 1.4. These results are attributed to the functionalities of Cs and GO. The functional groups interacted with the charged component of the bacterial surface membrane (i.e., phospholipids and lipopolysaccharides) [39], which interrupted the bacterial activity of both pathogens. CS has advantages over other polysaccharides due to the –NH_2_ functionality that easily bonds with bacterial DNA. That is one of the reasons that an increasing amount of CS causes more antibacterial activities. By forming new bonds with bacterial DNA, hydrogel controls the whole bacteria and hinders its further growth. Due to their sharp edges, GO nanosheets rapture the bacterial membranes and their functional groups and control the bacterial activities. Hence, an increasing amount of GO caused even better antibacterial activities than CS [22].

### 4.8. Cell Morphology

**Figure 11:**
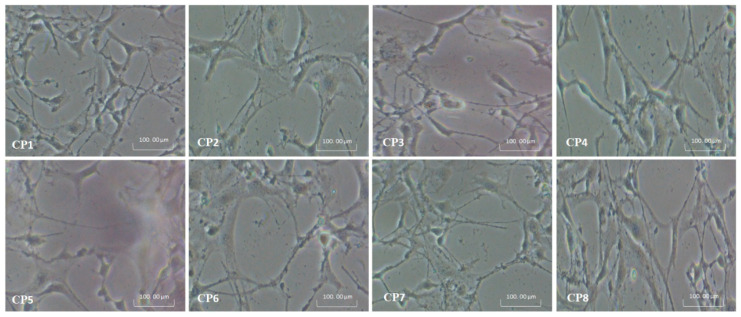
Shows the cell morphology of all hydrogels against pre-osteoblast (MC3T3-E1) cell lines.

*MC3T3-E1* cell lines were used to evaluate the in-vitro biocompatibility of hydrogels. The results are presented in Figure 11. Different functional groups enhance biocompatibility and cell differentiation on the substrate surfaces [28]. The increasing amount of CS causes more cell growth rather than an increasing amount of PVA (CP1 > CP2 > CP4 > CP3 > CP5 > CP6) due to the biocompatible affinity of CS with the MC3T3-E1 cell lines. On the other hand, an increasing GO facilitates H-bonding (CP8 > CP7) due to its various functional groups. The increasing amount of GO increases the functionalities and surface area that helps cell adherence and cell growth. It, in turn, confirms the enhancement of the micro-environment that expedites extracellular matrix (ECM) and biomaterials [40]. The matrix of the composite hydrogels has different chargeable functional groups (–COOH, –NH_2_, –H and –OH groups), and hydrogels have several active sites for cell adhesion, growth and spreading. Protein adsorption modulation via integrin binding to negative modified surfaces could be used to regulate cell adhesion. The fibronectin adsorption and integrin-binding have been reported in the following order –OH > –COOH > –NH_2_ > –CH_3_ to enhance osteoblasts cells [41]. 

### 4.9. Cell Viability and Optical Density

**Figure 12:**
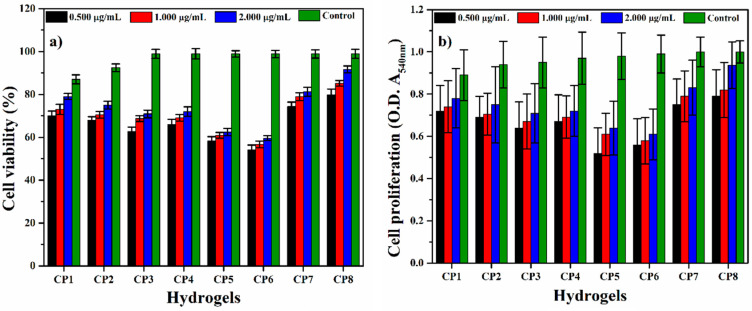
(**a**) cell viability and (**b**) optical density of all hydrogels against pre-osteoblast (*MC3T3-E1*) cell lines.

The cell viability and optical density assay of hydrogel samples were observed against *MC3T3-E1* cell lines with alongside concentrations (0.500, 1.000 and 2.000 µg/mL) after 72 h of incubation at 37 °C [42,43] as shown in Figure 12a,b. Among all hydrogels, CP1 and CP2, CP7 and CP8 exhibited maximum cell viability and optical density. Consequently, concentration 3 µg/mL was more appropriate for better results [2,43]. It was found that increasing the concentration of CS facilitates cell viability and optical density. Furthermore, a similar effect is observed with the increase in the amount of GO. As mentioned, these results were also attributed to the biocompatible affinity of CS with the *MC3T3-E1* cell lines and enhance the micro-environment (that expedite extracellular matrix (*ECM*)) provided by the functionalities of GO. Hence, all hydrogels had cell viability and nontoxicity towards pre-osteoblast cells with different values.

## 5. Conclusions

In the present work, novel hydrogels were successfully fabricated using the solution casting method. The best composition was selected and crosslinked with a critical concentration of tetraethyl orthosilicate (TEOS). For the fabrication of the GO/CS/PVA composite, this mixture was used. The samples were also tested for their ability to swell in a variety of media. The degradation analysis reveals that CP8 has a delayed degradation pattern, whereas CP1 has a rapid degradation behavior. The drug release profile was studied in phosphate-buffered saline (PBS) at a pH of 7.4. The data were fitted into kinetic models to predict the mechanism of drug release. Finally, information on antibacterial activity and cell viability was obtained. The successful synthesis of CS/PVA hydrogels and GO/CS/PVA composite hydrogel was revealed by FTIR studies. SEM revealed no phase separation between the polymers, whereas AFM revealed decreased surface roughness as GO content increased. The critical crosslinker concentration was 100 µL, at which the sample showed excellent swelling and structure preservation. The crosslinked and composite hydrogels both swelled, showing different swelling behavior. Diffusion-controlled drug release is the most acceptable mechanism, and it follows Fick’s law of diffusion for drug release. Our hypothesis was supported by the best fitting zero-order, Hixson-Crowell and Higuchi models. The antibacterial and cell viability properties of the GO/CS/PVA hydrogels composite were better wound dressing biomaterial for wound healing and treatment applications.

## Figures and Tables

**Table 1:** Formulations used for the preparation of hydrogels.

Samples	Formulation	CS (%)	PVA (%)	GO (%)	TEOS (µL)
**CP 1**	CS/PVA	80	20	0.00	100
**CP 2**	CS/PVA	60	40	0.00	100
**CP 3**	CS/PVA	20	80	0.00	100
**CP 4**	CS/PVA	40	60	0.00	100
**CP 5**	CS/PVA	60	40	0.00	0
**CP 6**	CS/PVA	60	40	0.00	150
**CP 7**	CS/PVA/GO-0.1%	60	40	0.1	100
**CP 8**	CS/PVA/GO-0.5%	60	40	0.5	100

**Table 2:** Avg. roughness of different hydrogel samples.

Sample	Avg. Roughness (nm)	Roughness (Centerline at Y-Axis) nm
**CP1 (a)**	2.401 ± 0.17	2.245 ± 0.19
**CP2 (c)**	3.861 ± 0.23	3.186 ± 0.27
**CP7 (e)**	4.644 ± 0.36	3.376 ± 0.43
**CP8 (g)**	7.532 ± 0.49	7.161 ± 0.57

## Data Availability

The data presented in this study are available on request from the corresponding author.
